# Students’ knowledge, attitude and practices towards pressure ulcer prevention and management

**DOI:** 10.4102/hsag.v28i0.2180

**Published:** 2023-01-26

**Authors:** Franco R. Abrahams, Edwin R. Daniels, Hileni N. Niikondo, Kristofina Amakali

**Affiliations:** 1 Department of General Nursing Sciences, Faculty of Health Sciences, School of Nursing, University of Namibia, Windhoek, Namibia; 2 Department of Allied Health, Faculty of Health Sciences, School of Nursing, University of Namibia, Windhoek, Namibia

**Keywords:** knowledge, attitudes, practices, student nurses, pressure ulcers

## Abstract

**Background:**

Student nurses provide nursing care to patients during clinical allocation, and their competence may affect the quality of care given to the patients. Good knowledge and positive attitudes enhance early detection for prevention and management of pressure ulcers.

**Aim:**

To determine undergraduate nursing students’ knowledge, attitude and practices (KAP) towards prevention and management of pressure ulcers.

**Setting:**

A nursing education institution in Windhoek, Namibia.

**Methods:**

A quantitative, cross-sectional research design was used to conveniently sample (*n* = 50) student nurses and collect data using a self-administered questionnaire. Data were analysed using the statistical software programme (SPSS) version 27. Descriptive frequencies were applied, and Fishers exact test was performed. A statistical value of *p* < 0.05 was considered significant.

**Results:**

Fifty (*n* = 50) student nurses consented to participate in the study. Student nurses reported good levels of knowledge (*n* = 35; 70%), attitude (*n* = 39; 78%), practices (*n* = 47; 94%). There was no statistically significant association between demographic variables and the level of knowledge, attitudes and practices, *p* > 0.05.

**Conclusion:**

Student nurses have good knowledge, positive attitudes and practices on prevention and management of pressure ulcers. By the implications, the study concludes that the nursing students will competently manage the pressure ulcers occurring in the clinical setting. An observational study is recommended to assess practices in the clinical setting.

**Contribution:**

The findings of this study will help to close the gap in the implementation of standard operating procedures for the prevention and management of pressure ulcers.

## Introduction

Preventing pressure ulcers should be the primary effort of all nurse managers in training hospitals in order to prevent chronic wounds that are difficult to manage and result in more complications. The competencies of undergraduate nursing students are essential for patient safety. This has been emphasised by Uba et al. (2015) and Kielo-Viljamaa et al. (2021). Therefore, Ebi, Hirko and Mijena (2019) emphasise that it is essential that nurses from the initial years of enrolment in the nursing training programme be oriented to the prevention and management of existing pressure ulcers presented by patients on admission. Internationally, the competencies of undergraduate nursing students have been a challenge that prompted high-income countries to develop instruments for measuring patient safety (Mortensen et al. 2022) to limit the worse outcome. However, Uba et al. (2015) and Lotfi et al. (2019) alluded that low-income and middle-income countries have delayed progress with moderate to low competence of nurses on prevention of pressure ulcers, notably because of limited exposure in the clinical practice.

According to Mekhoa, Mooi and Baloyi (2022), pressure ulcers are still a significant problem globally, but the lower and middle-income countries are more affected by pressure ulcers because of poor health systems. Lotfi et al. (2019) and Van Damme et al. (2019) argue that the presence or absence of pressure ulcers illustrates the quality of nursing care provided by the healthcare workers.

Namibia’s undergraduate nursing training curriculum emphasises competencies on patient safety; however, the application of the knowledge into practice by unexposed undergraduates is noted to be a challenge because of a lack of evidence-based outcomes. The lack of evidence prompted the authors to conduct the study to assess undergraduate nursing students’ knowledge, attitudes and practices towards pressure ulcer prevention and management.

### Nurses’ knowledge of pressure ulcers

Insufficient knowledge and skills, and negative attitudes towards pressure ulcer prevention may lead to the worsening of pressure ulcers (Dalvand, Ebadi & Gheshlagh 2018; Fernandes, Lima & Santos 2021). Therefore, Ortega et al. (2020) and Fernandes et al. (2021) warn that the development of pressure ulcers in the hospital may lead to medico-legal hazards resulting in litigation because of a lack of care. Most developed (high-income) countries hire competent nurses to facilitate quality patient care and secure their market (Bahrambeygi et al. 2019; Ortega et al. 2020). In lower-income countries, the prevalence of a lack of knowledge on ulcer is noted as evidenced by incompetence among nurses especially in managing pressure ulcers (Dalvand et al. 2018; Furtado et al. 2022).

### Nurses’ attitude towards pressure ulcers

Poor attitudes regarding pressure ulcers and negative feelings towards providing care to patients with pressure ulcers as well as their preferences in terms of wound management may lead to the development of pressure ulcers (Sucu & Kilic 2022). The authors furthermore indicated that positive attitude among nurses may lead to wound improvement and create satisfaction while dealing with pressure ulcers. On the contrary, Du et al. (2021) and Sucu and Kilic (2022) argue that negative attitude among nurses who dislike dealing with pressure wounds may result in complications and prolonged healing of patients. Furthermore, the authors expounded that attitudes influence the practice of risk assessment associated with pressure ulcers to identify possibilities and barriers in prevention and treatment of pressure ulcers.

### Nurses’ practices towards pressure ulcers

Similarly, practice is reflected in the psychomotor domain as the student nurse applies the theory to practice by implying high-quality care to prevent pressure ulcers development (Moura et al. 2020; Sucu & Kilic 2022). Early detection, prevention and treatment are particularly important for the successful management of pressure ulcers (Boyko, Longaker & Yang 2018; Furtado et al. 2022). According to Bahrambeygi et al. (2019), the effects of pressure ulcers delay rehabilitation, and contribute to prolonged morbidity and time of discharge, which raises healthcare costs because of the need for more resources and nursing hours while untreated ulcers may contribute to disability and death. Internationally, Dalvand et al. (2018) and Bahrambeygi et al. (2019) indicated that poor nurses’ knowledge on the prevention and management of pressure ulcers does not comply with practical guidelines, which are considered instruments for knowledge improvement. In Namibia, the occurrences of pressure ulcers are mostly poorly documented because of a lack of awareness among healthcare workers. Therefore, data on the knowledge, attitudes and practice of pressure ulcers among undergraduate students were not available at public health hospitals.

Pressure ulcers are preventable when correct assessment, planning and care are provided (Dalvand et al. 2018; Moura et al. 2020). Classification of pressure ulcers is necessary to determine the right intervention and to enhance recovery. It is necessary to develop the curriculum content with essential information and application of knowledge on nutrition, assessment, uses of barrier creams, patient positioning and education to patient and relative as major interventions in prevention and management of pressure ulcers (Kielo et al. 2020; Furtado et al. 2022). However, high workload is one of the factors leading to a lack of patients re-positioned as needed, thereby resulting in the development of pressure ulcers (Berihu et al. 2020; Ebi et al. 2019; Reynolds 2008).

### Problem statement

Limited and outdated guidelines, contained in old course outlines and books regarding pressure ulcer prevention are observed at public training hospitals in Windhoek where the participants of this study do their clinical practice. Studies indicated that teaching of pressure ulcer management and prevention from 1st year to student nurses provides the knowledge on skincare, nutrition, mechanical loading and management of existing ulcers (Fernandes et al. 2022; Isa et al. 2019; Lotfi et al. 2019). Post registration education in this domain leads to better knowledge than those who did not go through in-service training (Du et al. 2021).

Possession of the knowledge of pressure ulcers by students indicates student nurses’ observance of the guidance of the *Namibian Nursing Act* (No. 8 of 2004) which requires that nurses promote the hygiene, protect patients’ skin, and promote physical comfort for patients in a view to facilitate healing (Namibian Nursing Council 2014).

Therefore, it is necessary for student nurses and professional nurses to apply theoretical knowledge in clinical settings to prevent the formation of pressure ulcers and the management of existing pressure ulcers.

However, because of a lack of evidence-based practice, in Namibia little is known about the nurses’ knowledge, attitude and practices regarding prevention and management of pressure ulcers. To the best of the authors’ knowledge, there is no published literature on pressure ulcers within the Namibian context. As students are among the nurses who provide care during clinical allocation, their competence may affect the quality of care given to the patients. Therefore, this study aimed to determine the knowledge, attitude and practices of student nurses on the prevention and management of pressure ulcers.

## Aim of the study

The aim of the study was to determine undergraduate nursing students’ knowledge, attitude and practices (KAP) towards the prevention and management of pressure ulcers.

The specific objectives of the study were:

- To determine the knowledge, attitudes and practices of undergraduate nursing students towards prevention and management of pressure ulcers.
- To describe the knowledge, attitudes and practices of undergraduate nursing students towards prevention and management of pressure ulcers.
- To assess the relationship between the demographic characteristic of undergraduate nursing students and their levels of knowledge, attitudes and practices towards pressure ulcer prevention and management.

## Research design and methods

### Research design

The study was a quantitative, cross-sectional and analytical study. This was a quantitative design to quantify and describe the knowledge, attitudes and practices towards pressure ulcer management and prevention and cross-sectional as the data were collected at one point in time. It was also an analytical study as it allowed the researchers to test the relationship between independent and dependent variables.

### Study setting

The study was conducted among the 4th year nursing students, at the School of Nursing at the University of Namibia (UNAM) main campus. The School of Nursing at UNAM has one main (urban) campus in Windhoek and three satellite (rural) campuses at Oshakati, Rundu and Keetmanshoop. Each campus hosts about 400 undergraduate nursing students across the different academic years, from the first to the 4th year of their studies. The School of Nursing offers the Bachelor of Nursing (Clinical) Honours degree. The programme is offered on a 2-week block system comprising both theory and practice. Students are expected to integrate theoretical thought concepts during clinical practice. Pressure ulcers prevention and management is an integral part of the subjects: General Nursing I and II. Therefore, 4th year nursing students are expected to have knowledge and skills regarding prevention and management of pressure ulcers, as they completed more than 1800 h of clinical practices for General Nursing Science.

### Study population and sampling strategy

At the time of the study, there were 73 (4th year) students registered at the University of Namibia’s main campus for the Bachelor of Nursing Science (Clinical) Honours degree.

The minimum sample required (*n* = 62) (at confidence interval of 95% and margin of error [*e*] of 5%) (Brink, Van der Walt & Van Rensburg 2012) was calculated using the Slovin’s formula where: sample size (*n*) = *N*/1+*Ne*^2^. A total of 50 out of the 62 students consented to participate in the study. A convenience sampling method was adopted for this study to select prospective participants from the 4th year nursing students at the main campus who were readily available to the researcher.

### Research instrument

A self-reported questionnaire adopted and modified from (Hommel & Santy-Tomlinson 2018; Mitchell 2018; NICE 2014; Tahvonen et al. 2017) was used to assess the knowledge, attitudes and practices regarding pressure ulcers among study participants. The questionnaire was composed in English only as this is the official medium of instruction at the university. The self-reported questionnaire comprised four sections. Section A was about the demographic data of the respondents. Section B was about respondents’ knowledge regarding pressure ulcers and required the respondents to indicate by selecting one of three options: either true, false or I don’t know to assess their knowledge on pressure ulcers. Section C contained 11 four-point Likert scale items (where 1 = strongly disagree, 2 = disagree, 3 = agree, 4 = strongly agree) that tested the attitude of student nurses towards prevention and management of pressure ulcer. Section D measured respondents’ practices regarding prevention and management of pressure ulcers. This section had 17 four-point Likert scale items (where 1 = Yes, I do it always, 2 = Yes sometimes, 3 = No I don’t, 4 = not at all). Cronbach’s alpha coefficients were calculated to test reliability of the research instrument. Cronbach’s alpha coefficients were acceptable for the sections of the questionnaire (knowledge scale = 0.56, attitude scale = 0.74 and practices scale = 0.79). The questionnaire was piloted with five 4th-year nursing students. The questionnaire was refined. The participants of the pilot study were excluded from the main study and the data from the pilot study were also excluded from the final data analysis.

### Data collection

#### Data collection procedure

Data were collected and supervised by the principal researcher in a class room at the School of Nursing, at the University of Namibia’s main campus. The data were collected on one day during November 2018, just before students wrote their final examination. Students were informed about the purpose, scope, benefits and risks associated with participation in the study. Fifty (*n* = 50) student nurses consented to participate in the study and signed informed consent. Each student was handed a questionnaire and reminded to drop it in the drop box provided. All questionnaires were coded to protect the identity of research respondents. In addition, no personal details of the respondents were recorded. Each questionnaire took approximately 30 min to complete. After completion, each participant dropped the completed questionnaire in the drop box provided. Upon completion of data collection, the researcher stored all completed questionnaires in a locked cabinet that was only accessible to the researcher.

#### Data analysis

The principal researcher examined questionnaires for completeness and entered data into Microsoft Excel version 16. The data were then transferred to the Statistical Package for the Social Sciences (SPSS) version 27 and descriptive statistics of measures of frequency were performed by a lecturer in the School of Nursing who teaches research methodology. Individual questionnaire items were scored, and overall scores were calculated. Findings were presented in tables of frequencies and percentages. The following criteria were applied.

For levels of knowledge: an overall score between 20 and 33 indicated poor knowledge, a score between 34 and 46 indicated satisfactory knowledge, and a score between 47 and 60 indicated good levels of knowledge. For levels of attitude: an overall score between 11 and 27 indicated poor attitude, a score between 28 and 44 indicated good attitude towards prevention of pressure ulcers. For levels of practices: an overall score between 0 and 5 indicated poor practices, a score between 6 and 11 indicated satisfactory practices, and a score between 11 and 17 indicated good practices. Fisher’s exact test was used to establish the association between the demographic variables and levels of knowledge, attitudes and practices. The results were considered statistically significant if a two-sided *p* < 0.05 was obtained.

### Ethical considerations

This research was conducted as part of course requirement for completion of the Bachelor of Nursing (Clinical) Honours degree and ethical approval for the study was granted by the Ethical Committee of the School of Nursing (Reference Number: SoN/N027/2018). Permission to conduct this study was sought and granted from the Chief Executive Officer of the Ministry of Health and Social Services. The purpose, aims and objectives of the study were explained to the respondents. Participation in the study was voluntary, and the respondents had the right to withdraw at any time without any repercussions. An information session was held with potential respondents.

## Results

A total of 50 respondents participated in the study and yielded 81% of sample return. The demographic characteristics of respondents were summarised by means of measures of frequency and are illustrated in Table 1. The association between the demographic data and the knowledge, attitudes and practices of the respondents was analysed by Fisher’s exact test considering statistical significance of *p* < 0.05.

**TABLE 1:** Demographic characteristics of respondents (*n* = 50).

Demographic characteristic	Frequency (*n*)	Percentage (%)
Total	50	100.0
**Age groups**		
21–30	47	94.0
31–40	3	6.0
**Gender**		
Male	6	12.0
Female	44	88.0
**Marital status**		
Single	44	88.0
Married	5	10.0
Other	1	2.0
**Length of time allocated in the ward**		
< a week	1	2.0
1–2 weeks	17	34.0
3–4 weeks	6	12.0
≤ 2 months	2	4.0
3–12 months	24	48.0
**Last time you listened to a lecture on pressure ulcer**		
≤ 1 year	22	44.0
> 1 year but < 2 years	14	28.0
2–3 years ago	7	14.0
Never	7	14.0
**Last time you read an article or book on pressure ulcers**		
≤ 1 year	17	34.0
> 1 year but < 2 years	14	28.0
2–3 years ago	10	20.0
Never	9	18.0

*Source:* Adapted from Hommel & Santy-Tomlinson 2018; Mitchell 2018; NICE 2014; Tahvonen et al. 2017

Note: Please see the full reference list of the article, Abrahams, F.R., Daniels, E.R., Niikondo, H.N. & Amakali, K., 2023, ‘Students’ knowledge, attitude and practices towards pressure ulcer prevention and management’, *Health SA Gesondheid* 28(0), a2180. https://doi.org/10.4102/hsag.v28i0.2180, for more information.

Table 1 shows that most of the respondents were females (*n* = 44; 88%), compared to males (*n* = 6; 12%). Only (*n* = 7; 14%) of the respondents did not receive training on pressure ulcers, while (*n* = 22; 44%) of the respondents received training recently (1 year or less). The table also displays that (*n* = 9; 18%) did not read books on pressure ulcers.

Table 2 shows the frequencies on knowledge on pressure ulcers. A total of (*n* = 36; 72%) of the respondents could correctly identify that pressure ulcers may be predisposed by dry or flaky skin. The table displays that (*n* = 15; 30%) of the respondents did not know that people who smoke are more susceptible to develop pressure ulcers and that smoking may slow the wound healing process of pressure ulcers. Most respondents (*n* = 34; 68%) did not know the assessment tools used to identify the risk for developing pressure ulcers.

**TABLE 2:** Frequencies on the knowledge of pressure ulcers (*n* = 50).

Knowledge characteristic	True	False	Don’t know
**Pressure ulcers are sterile wounds**			
Frequency (*n*)	6	41	3
Percentage (%)	12.0	82.0	6.0
**Dragging the patient up in bed increases friction**			
Frequency (*n*)	46	2	2
Percentage (%)	92.0	4.0	4.0
**Overweight patients present more handling difficulties and shearing force, and friction can be particularly problematic when moving such patients**			
Frequency (*n*)	46	-	4
Percentage (%)	92.0	-	8.0
**Obese patients have reduced tissue oxygenation and collagen production which in turn will slow healing of the existing pressure ulcer**			
Frequency (*n*)	37	2	11
Percentage (%)	74.0	4.0	22.0
**Many underlying conditions or disease can cause immobility, loss of sensation, excessive perspiration, muscle spasms and incontinence predisposing patients to develop pressure ulcers**			
Frequency (*n*)	43	2	5
Percentage (%)	86.0	4.0	10.0
**Research has also linked dry flaky or scaling skin to an increased incident of pressure ulcers**			
Frequency (*n*)	36	3	11
Percentage (%)	72.0	6.00	22.0
**Research has reported that being male increases the risk of developing a pressure ulcer by 86%**			
Frequency (*n*)	2	19	29
Percentage (%)	4.0	38.0	58.0
**Smoking can also contribute to the risk of pressure ulcers development; therefore, it can also have a negative influence on the healing of existing pressure ulcer**			
Frequency (*n*)	27	8	15
Percentage (%)	54.0	16.0	30.0
**Any pressure point is a vulnerable area when pressure is intense and prolonged**			
Frequency (*n*)	45	2	3
Percentage (%)	90.0	4.0	6.0
**At risk pressure point in the sitting position is heels and toes, ischial tuberosity, sacrum, scapular and occiput**			
Frequency (*n*)	42	5	3
Percentage (%)	84.0	10.0	6.0
**Pressure ulcers are caused by unrelieved pressure shear, friction**			
Frequency (*n*)	49	1	-
Percentage (%)	98.0	2.0	-
**Although pressure ulcers can occur at any age, there is a correlation between ageing process and the incident of pressure ulcer**			
Frequency (*n*)	42	1	7
Percentage (%)	84.0	2.0	14.0
**Many conditions such as stroke, spinal cord trauma, head injury, over sedation, depression and confusion contribute to immobility. It predisposes to shearing and friction and is associated with development of larger ulcers**			
Frequency (*n*)	43	3	4
Percentage (%)	86.0	6.0	8.0
**Some drugs that cause diarrhoea or urinary incontinence can add to the risk of pressure ulcer development**			
Frequency (*n*)	17	10	23
Percentage (%)	34.0	20.0	46.0
**It has been suggested that faecal incontinence may be a more important risk factor than urinary incontinence**			
Frequency (*n*)	8	12	30
Percentage (%)	16.0	24.0	60.0
**Over 2 million people develop pressure ulcers a year worldwide**			
Frequency (*n*)	12	2	36
Percentage (%)	24.0	4.0	72.0
**An essential aspect of pressure ulcer prevention is identification of those at risk**			
Frequency (*n*)	46	1	3
Percentage (%)	92.0	2.0	6.0
**A pressure ulcer assessment scale attempts to identify the presence of extrinsic and intrinsic actors that cause pressure ulcers**			
Frequency (*n*)	33	6	11
Percentage (%)	66.0	12.0	22.0
**Bed or chair bound individuals or those whose ability is impaired should be considered at risk for pressure ulcer development**			
Frequency (*n*)	39	6	5
Percentage (%)	78.0	12.0	10.0
**The Norton, Waterlow and Brandon scale are all risk assessment tools**			
Frequency (*n*)	11	5	34
Percentage (%)	22.0	10.0	68.0

*Source:* Adapted from Hommel & Santy-Tomlinson 2018; Mitchell 2018; NICE 2014; Tahvonen et al. 2017

Note: Please see the full reference list of the article, Abrahams, F.R., Daniels, E.R., Niikondo, H.N. & Amakali, K., 2023, ‘Students’ knowledge, attitude and practices towards pressure ulcer prevention and management’, *Health SA Gesondheid* 28(0), a2180. https://doi.org/10.4102/hsag.v28i0.2180, for more information.

Table 3 shows that (*n* = 33; 66%) of the respondents agreed that pressure ulcers may cause patient mortality. When respondents were asked to indicate whether daily hygiene such as bathing may prevent ulcers, only (*n* = 4; 8%) strongly agreed to this statement while (*n* = 10; 20%) disagreed. A total of (*n* = 26; 52%) of the respondents agreed that pressure ulcers can be prevented by massaging bony prominences in order to enhance blood circulation, while (*n* = 4; 8%) strongly disagreed with this statement. Only (*n* = 10; 20%) of the respondents strongly agreed that it is expensive to treat pressure ulcers while (*n* = 12; 24%) strongly disagreed with this statement.

**TABLE 3:** Frequencies on the attitudes to pressure ulcers (*n* = 50).

Attitude characteristic	Strongly disagree	Disagree	Agree	Strongly agree
**Do you believe a pressure ulcer can lead to death?**				
Frequency (*n*)	5	9	33	3
Percentage (%)	10.0	18.0	66.0	6.0
**Obese patients are rarely malnourished and therefore at lower risk of developing pressure ulcers.**				
Frequency (*n*)	16	24	10	-
Percentage (%)	32.0	48.0	20.0	-
**Do you believe a daily bath or sponge bath will prevent pressure ulcers?**				
Frequency (*n*)	3	10	33	4
Percentage (%)	6.0	20.0	66.0	8.0
**Do you believe friction and shear may occur when sliding the person up in bed?**				
Frequency (*n*)	3	7	27	13
Percentage (%)	6.0	14.0	54.0	26.0
**Do you believe a blister on a patient heel is not of concern?**				
Frequency (*n*)	20	24	5	1
Percentage (%)	40.0	48.0	10.0	2.0
**Erythema or redness on any patient that is not blanchable should be documented and reported.**				
Frequency (*n*)	5	3	24	18
Percentage (%)	10.0	6.0	48.0	36.0
**Bony prominences should not have direct contact with one another.**				
Frequency (*n*)	3	5	25	17
Percentage (%)	6.0	10.0	50.0	34.0
**Massaging a bony prominence promotes circulation and prevent pressure ulcers.**				
Frequency (*n*)	4	6	26	14
Percentage (%)	8.0	12.0	52.0	28.0
**Do you believe patients who are immobile, orthopaedic, decreased mental status, unconscious are at elevated risk of developing pressure ulcers?**				
Frequency (*n*)	3	4	21	22
Percentage (%)	6.0	8.0	42.0	44.0
**Pressure ulcers are a painful condition among people who are elderly or physically impaired.**				
Frequency (*n*)	3	9	21	17
Percentage (%)	6.0	18.0	42.0	34.0
**Do you believe pressure ulcers are very costly to treat?**				
Frequency (*n*)	12	14	14	10
Percentage (%)	24.0	28.0	28.0	20.0

*Source:* Adapted from Hommel & Santy-Tomlinson 2018; Mitchell 2018; NICE 2014; Tahvonen et al. 2017

Note: Please see the full reference list of the article, Abrahams, F.R., Daniels, E.R., Niikondo, H.N. & Amakali, K., 2023, ‘Students’ knowledge, attitude and practices towards pressure ulcer prevention and management’, Health SA Gesondheid 28(0), a2180. https://doi.org/10.4102/hsag.v28i0.2180, for more information.

Table 4 shows that (*n* = 29; 58%) of the respondents conduct systematic skin inspections at least once a day, and record the results, while only (*n* = 3; 6%) do not practice systematic skin inspections at least once a day. The table also indicates that (*n* = 11; 22%) of respondents change their patient’s position at 15-min intervals, while (*n* = 19; 38%) will only change their patient’s positions occasionally. Thirteen (26%) did not practise this routine recommendation at all. From the total respondents who participated, a total of (*n* = 41; 82%) indicated that they provide the patient and their care givers (support structures) with information on methods on how to prevent pressure ulcers, while only (*n* = 2; 4%) do not provide information to their patients or care givers.

**TABLE 4:** Frequencies on the practices on pressure ulcer prevention (*n* = 50).

Practices characteristic	Yes, I do it always	Yes sometimes	No, I don’t	Not at all
**All individuals at risk should have a systematic skin inspection at least once a day, paying particular attention to the bony prominences and the results of the skin inspection should be documented**				
Frequency (*n*)	29	18	3	-
Percentage (%)	58.0	36.0	6.0	-
**Individuals in bed who are completely immobile should have a care plan that includes the use of devices that totally relieve pressure on the heel, most commonly by raising the heels off the bed. Do not use doughnut-type devices**				
Frequency (*n*)	28	17	5	-
Percentage (%)	56.0	34.0	10.0	-
**Do you use linen savers, under pads, briefs that can absorb moisture and which can present a quick drying surface to protect the skin against moisture**				
Frequency (*n*)	31	16	2	1
Percentage (%)	62.0	32.0	4.0	2.0
**The frequency of reassessment depends on patient status and institutional policy**				
Frequency (*n*)	20	16	8	6
Percentage (%)	40.0	32.0	16.0	12.0
**For bed bounded individuals, use devices that totally relieve pressure on the heels**				
Frequency (*n*)	27	14	6	3
Percentage (%)	54.0	28.0	12.0	6.0
**If the pressure ulcer is infected, obtain a sample of the drainage for culture and sensitivity to antiseptic agents**				
Frequency (*n*)	25	19	5	1
Percentage (%)	50.0	38.0	10.0	2.0
**For patients at risk, the nurse must avoid massage over bony prominences as current evidence suggests that this may be harmful.**				
Frequency (*n*)	10	19	12	9
Percentage (%)	20.0	38.0	24.0	18.0
**For bed bound individuals, place at risk patients on a pressure reducing mattress. Do not use doughnut-type devices**				
Frequency (*n*)	19	14	14	3
Percentage (%)	38.0	28.0	28.0	6.0
**For chair bound individuals, patients must have an individualised bathing schedule, avoid hot water, use a mild cleaning agent**				
Frequency (*n*)	22	17	10	1
Percentage (%)	44.0	34.0	20.0	2.0
**Repositioning must be done at least every 15 min**				
Frequency (*n*)	11	19	7	13
Percentage (%)	22.0	38.0	14.0	26.0
**Do you inspect pressure areas for abrasions and excoriations**				
Frequency (*n*)	37	9	3	1
Percentage (%)	74.0	18.0	6.0	2.0
**Providing the patient with a smooth, firm, and wrinkle-free foundation on which to sit or lie helps prevent skin trauma**				
Frequency (*n*)	34	13	2	1
Percentage (%)	68.0	26.0	4.0	2.0
**When lifting the patient to a changing position, nurses should use a lifting device such as a trapeze rather than dragging the patient across or up in bed**				
Frequency (*n*)	26	15	6	3
Percentage (%)	52.0	30.0	12.0	6.0
**Reducing shear force by keeping the head of the bed flat or elevated to a maximum of 30°, unless contraindicated by the patient’s condition**				
Frequency (*n*)	23	20	5	2
Percentage (%)	46.0	40.0	10.0	4.0
**Encouraging the patient to change position frequently even if only slightly**				
Frequency (*n*)	35	10	4	1
Percentage (%)	70.0	20.0	8.0	2.0
**Encourage the patient to do active range-of-motion (ROM) exercises every 2–3 h**				
Frequency (*n*)	26	15	5	4
Percentage (%)	52.0	30.0	10.0	8.0
**Teaching the patient and support persons self-care measures to prevent pressure ulcers**				
Frequency (*n*)	41	5	2	2
Percentage (%)	82.0	10.0	4.0	4.0

*Source:* Adapted from Hommel & Santy-Tomlinson 2018; Mitchell 2018; NICE 2014; Tahvonen et al. 2017

Note: Please see the full reference list of the article, Abrahams, F.R., Daniels, E.R., Niikondo, H.N. & Amakali, K., 2023, ‘Students’ knowledge, attitude and practices towards pressure ulcer prevention and management’, Health SA Gesondheid 28(0), a2180. https://doi.org/10.4102/hsag.v28i0.2180, for more information.

The overall knowledge, attitude and practices of each participant were calculated and are presented in Table 5.

**TABLE 5:** Frequencies of levels of knowledge, attitudes and practices of respondents (*n* = 50).

Variables	Overall KAP levels													
	Knowledge						Attitude				Practices			
	Poor		Satisfactory		Good		Poor		Good		Poor		Good	
		
	*N*	%	*N*	%	*N*	%	*N*	%	*N*	%	*N*	%	*N*	%
Total	0	0	15	30	35	70	11	22	39	78	3	6	47	94
**Age group**														
21–30	0	0	14	29.8	33	70.2	10	21.3	37	78.7	3	6.4	44	93.6
31–40	0	0	1	33.3	2	66.7	1	33.3	2	66.7	0	0	3	100
*P*	1.000						0.534				1.000			
**Gender**														
Male	0	0	0	0	6	100	0	0	6	100	0	0	6	100
Female	0	0	15	34.1	29	65.9	11	25	33	75	3	6.8	41	93.2
*P*			0.160						0.317				1.000	
**Marital status**														
Single	0	0	13	29.5	31	70.5	9	20.5	35	79.5	3	6.8	41	93.2
Married	0	0	1	20	4	80	1	20	4	80	0	0	5	100
Other	0	0	1	100	0	0	1	100	0	0	0	0	1	100
*P*	0.276						0.164				0.804			
**Length allocated in the ward**														
1–2 weeks	0	0	7	38.9	11	61.1	4	22.2	14	77.8	2	11.1	16	88.9
3–4 weeks	0	0	3	50	3	50	0	0	6	100	1	16.7	5	83.3
≤ 2 months	0	0	0	0	2	100	0	0	2	100	0	0	2	100
3–12 months	0	0	5	20.8	19	79.2	7	29.2	17	70.8	0	0	24	100
*P*	0.303						0.395				0.295			
**Last time you listened to a lecture on pressure ulcer**														
≤ 1 year	0	0	3	13.65	19	86.4	3	16.6	19	86.4	2	9.1	20	90.9
> 1 year but < 2 years	0	0	6	42.9	8	57.1	6	42.9	8	57.1	1	7.1	13	92.9
2–3 years ago	0	0	3	42.9	4	57.1	1	14.3	6	85.7	0	0	7	100
Never	0	0	3	42.9	4	57.1	1	14.3	6	65.7	0	0	7	100
*P*	0.171						0.177				0.729			
**Last time you read an article on pressure ulcers**														
≤ 1 year	0	0	4	23.5	13	76.5	4	23.5	13	76.5	1	5.9	16	94.1
> 1 year but < 2 years	0	0	2	14.3	12	85.7	4	28.6	10	71.4	0	0	14	100
2–3 years ago	0	0	5	50	5	50	2	20	8	80	2	20	8	80
Never	0	0	4	44.4	5	55.6	1	11.1	8	88.9	0	0	9	100
*P*	0.188						0.796				0.176			

*Source:* Adapted from Hommel & Santy-Tomlinson 2018; Mitchell 2018; NICE 2014; Tahvonen et al. 2017

Note: Please see the full reference list of the article, Abrahams, F.R., Daniels, E.R., Niikondo, H.N. & Amakali, K., 2023, ‘Students’ knowledge, attitude and practices towards pressure ulcer prevention and management’, *Health SA Gesondheid* 28(0), a2180. https://doi.org/10.4102/hsag.v28i0.2180, for more information.

KAP, knowledge, attitude and practices.

Significance of Fisher’s exact test (*p* < 0.05).

Table 5 gives a descriptive summary of the knowledge, attitude and practice scores of the respondents. Thirty-five (70%) of the respondents had good knowledge, (*n* = 39; 78%) a good attitude and (*n* = 47; 94%) good practices towards pressure ulcer management and prevention. The Fisher’s exact test was to correlate the participant’s demographics with the participant’s overall knowledge, attitude and practices scores (Table 5). There was no statistically significant association between respondents’ demographic information and overall KAP scores (*p* > 0.05).

## Discussion

This study assessed nursing students’ knowledge, attitudes and practices towards prevention and management of pressure ulcers.

The results of the study showed that majority (94%) of the respondents were female compared to males. These results are consistent with the literature, which indicates that the nursing profession is female-dominated, mainly because society compartmentalises and considers nursing as a female profession (Stanley et al. 2016). The result of the current study is not incidental but reflects the actual number of males admitted into the nursing profession in Namibia. Equally, the majority (94%) of the respondents were young (21–30 years), which indicated that they were novices to the nursing profession. Therefore, as a prospective nurse practitioner, it was important to assess student nurses’ knowledge, attitudes and practices regarding prevention and management of pressure ulcer.

### Student nurses’ knowledge of pressure ulcers

Nurses’ knowledge about prevention and management of pressure ulcers was assessed. The findings indicate that the majority (70%) of respondents had good knowledge about the causes and prevention of pressure ulcers respectively and only 30% had satisfactory knowledge. The findings in this study contrast with Murugiah et al. (2020) and Isa, Azman and Mat (2019) who reported that student nurses portray low levels of knowledge concerning pressure ulcers. However, the findings of 30% of the respondents from this study, who demonstrated only satisfactory knowledge is of concern, because they would put patients at risk. A concern for risk to patients owing to a lack of knowledge of pressure ulcers concurs with the reports by Dalvand et al. (2018) and Furtado et al. (2022) who indicated that ulcer development is associated with a lack of knowledge and incompetence in managing pressure ulcers among nurses.

De Meyer et al. (2019) expounded that the knowledge of pressure ulcers may increase with higher educational levels or training on pressure ulcers. In support of the increase of knowledge of pressure ulcers, the findings from this study indicated that respondents who are in the age category of 31–40 years, who by implications could be previously enrolled nurses demonstrated 100% knowledge level about pressure ulcers.

The findings of this study support the suggestions by Claudia et al. (2010) and Bollineni (2019) who assert that student nurses as prospective qualified nurses should possess the knowledge on pressure ulcers and be conversant in methods for prevention and management of pressure ulcers. Possession of the knowledge of pressure ulcers by students indicates student nurses’ observance of the guidance of the *Namibian Nursing Act* (No. 8 of 2004) which requires that nurses promote the hygiene, protect patients’ skin and promote physical comfort for patients in a view to facilitate healing (Namibian Nursing Council 2014). Otherwise, where the knowledge is lacking, Malinga and Dlungwane (2020) recommend training to improve the knowledge of student nurses regarding prevention and management of pressure ulcers. In their study, Isa et al. (2019) reported that students with high levels of knowledge on pressure ulcers will display a positive attitude towards prevention and management of pressure ulcers.

### Student nurses’ attitude towards pressure ulcers

Student nurses’ attitudes towards prevention and management of pressure ulcers were determined to describe distinctive characteristic which regulates the respondents’ actions towards prevention and management of pressure ulcers. The findings indicated that more than a third (78%) of the respondents in this study had a good or positive attitude towards the prevention and management of pressure ulcers. The findings of this study are congruent with the reports of Uba et al. (2015) and Isa et al. (2019) which indicate that student nurses had a high positive attitude towards prevention of pressure ulcers. Isa et al. (2019) support the findings of this study regarding possession of higher knowledge of pressure ulcers that is translated into a positive attitude towards the prevention of pressure ulcers.

According to Simonetti et al. (2015), clinical competence may be correlated with the extent of clinical exposure thereby increasing the student’s confidence and positive attitudes towards care. Similarly, in the Namibian context, student nurses are first taught and only allowed to perform nursing procedures on previously simulated content by their lecturers for them to acquire and practise nursing skills appropriately (Mukumbang & Adejumo 2014). Therefore, the findings of positive attitude towards prevention of pressure ulcers among the respondents of the current study may indicate the inculcations of appropriate attitudes towards care during simulation training and nurtured during the course of their studies.

Nevertheless, the findings of this study do not indicate a statistical significance between the respondents’ positive attitude and the last time respondents listened to a lecture on pressure ulcers (*p* = 0.177) or the last time the respondents read an article or book on pressure ulcers (*p* = 0.796). Good level of knowledge about and positive attitudes towards prevention and management of pressure ulcers would result in good practice regarding the prevention and management of pressure ulcers.

### Student nurses’ practices towards the prevention and management of pressure ulcers

The findings indicated that majority (94%) of the nursing students in this study had good practice towards prevention of pressure ulcers. Good practice is a result of good knowledge and positive attitudes towards prevention and management of pressure ulcers as alluded on in the previous sections. The findings of this study concur with the report of Sham et al. (2020) which indicated that nurses demonstrated a good practice (96.8%) towards prevention and management of pressure ulcers, but not in accord with the findings of a study conducted in Guntur which only indicates slightly more than half (51.1%) of the respondents who had satisfactory practices compared to (48.9%) who had unsatisfactory practices. However, the finding of current study still raises concern as it indicated that only a few (22%) of the respondents practised changing of their patients’ positions at 15-min intervals, a significant (38%) only change their patients’ positions occasionally, while (26%) did not practise this routine recommendation for prevention of pressure ulcers. It can therefore be concluded that although students have good knowledge, patients may still be at risk of developing pressure ulcers if students do not change their patients’ positions at required intervals.

The relationship between demographic factors and respondents’ practices was also assessed. No statistical significance was found between the last time students listened to a lecture on pressure ulcers (*p* = 0.729) or the last time students read an article or book on pressure ulcers (*p* = 0.176) and student levels of practices to pressure ulcers.

### Study limitations

The study was limited to student nurses at one public university in Windhoek, Namibia. The study did not include nursing students from other training institutions or satellite campuses of the aforementioned university. The study findings cannot be generalised because a non-probability convenient sampling technique was used resulting in a small sample size which was less than the required sample size indicated by the calculated formula. Although nursing students reported good level of knowledge, attitudes and practices, data were collected with a self-administered questionnaire which may be prone to bias. This was however controlled by providing a space where respondents could complete the questionnaires without any interaction with each other or search the internet for answers. This study does not address the practical component of respondents and therefore practices reported by students cannot be guaranteed.

## Conclusion

In conclusion, the findings of this study suggest that student nurses have good knowledge, positive attitudes and practices on prevention and management of pressure ulcers. Although the study showed that student nurses have good knowledge, the findings raise concern as a significant portion (30%) of the respondents had no knowledge that people who smoke are predisposed to the development of pressure ulcers and slows wound healing. Furthermore, the findings indicated that the majority (78%) of the respondents lacked knowledge on the different tools that are used to assess and identify patients at risk for developing pressure ulcers. By the implications, the study concludes that the nursing students will competently manage the pressure ulcers occurring in the clinical setting. The authors, therefore, recommend reinforcement of risk factors and tools for pressure ulcer development and management in student curricula towards the final year of study.
